# Cross-sectional study into age-related pathology of mouse models for limb girdle muscular dystrophy types 2D and 2F

**DOI:** 10.1371/journal.pone.0220665

**Published:** 2019-08-20

**Authors:** Ingrid E. C. Verhaart, Kayleigh Putker, Davy van de Vijver, Christa L. Tanganyika-de Winter, Svetlana Pasteuning-Vuhman, Jaap J. Plomp, Annemieke M. Aartsma-Rus, Maaike van Putten

**Affiliations:** 1 Department of Human Genetics, Leiden University Medical Center, Leiden, the Netherlands; 2 Department of Neurology, Leiden University Medical Center, Leiden, the Netherlands; University of Houston, UNITED STATES

## Abstract

Limb girdle muscular dystrophy (LGMD) types 2D and 2F are caused by mutations in the genes encoding for α- and δ-sarcoglycan, respectively, leading to progressive muscle weakness. Mouse models exist for LGMD2D (*Sgca*^*-/-*^) and 2F (*Sgcd*^*-/-*^). In a previous natural history study, we described the pathology in these mice at 34 weeks of age. However, the development of muscle pathology at younger ages has not been fully characterised yet. We therefore performed a study into age-related changes in muscle function and pathology by examining mice at different ages. From 4 weeks of age onwards, male mice were subjected to functional tests and sacrificed at respectively 8, 16 or 24 weeks of age. Muscle histopathology and expression of genes involved in muscle pathology were analysed for several skeletal muscles, while miRNA levels were assessed in serum. In addition, for *Sgcd*^*-/-*^ mice heart pathology was assessed. Muscle function showed a gradual decline in both *Sgca*^*-/-*^ and *Sgcd*^*-/-*^ mice. Respiratory function was also impaired at all examined timepoints. Already at 8 weeks of age, muscle pathology was prominent, and fibrotic, inflammatory and regenerative markers were elevated, which remained relatively constant with age. In addition, *Sgcd*^*-/-*^ mice showed signs of cardiomyopathy from 16 weeks of age onwards. These results indicate that *Sgca*^*-/-*^ and *Sgcd*^*-/-*^ are relevant disease models for LGMD2D and 2F.

## Introduction

The limb girdle muscular dystrophies (LGMDs) comprise the most heterogeneous collection of muscular dystrophies with over 30 subtypes known. They are identified according to their genetic defects with autosomal dominantly and recessively inherited LGMDs sub-grouped as LGMD1 and LGMD2, respectively. LGMDs are characterised by a progressive weakness of proximal muscles of the hip and shoulder girdles [1]. Sarcoglycanopathies comprise four subtypes, LGMD2C, -D, -E and -F, which form the more common variants of LGMD. They are caused by mutations in the genes coding for the muscle-specific transmembrane sarcoglycan proteins α-, β-, γ-, and δ-sarcoglycan [2]. Sarcoglycans are crucial components of the dystrophin-glycoprotein complex that physically connects the intracellular cytoskeleton to the extracellular matrix. The loss of this structural linkage, for instance due to mutations in one of the sarcoglycan genes, makes muscle fibres more susceptible to damage during muscle contractions [3–6]. Although causative gene mutations are well known, there is currently no specific therapy available for sarcoglycanopathies [7].

Animal models for α- and δ-sarcoglycanopathies [B6.129S6-Sgca^tm2Kcam^/J (*Sgca*^*-/-*^; LGMD2D) and B6.129-Sgcd^tm1Mcn^/J (*Sgcd*^*-/-*^; LGMD2F) mice, respectively] have been generated mimicking many features of the human disease [8, 9]. We previously performed a comprehensive longitudinal natural disease history study in which we assessed muscle function in *Sgca*^*-/-*^ and *Sgcd*^*-/-*^ mice for 30 weeks (from 4 to 34 weeks of age) on a bi-weekly basis [10]. Although this study was instrumental for setting up standardized pre-clinical trials in LGMD2D and 2F mice, direct comparisons between muscle function and pathology at younger ages could not be made. Therefore, we here present a cross-sectional study in *Sgca*^*-/-*^ and *Sgcd*^*-/-*^ mice in which we correlate muscle function and pathology at 8, 16 and 24 weeks of age. This study provides a comprehensive insight in the age-related development of pathology in *Sgca*^*-/-*^ and *Sgcd*^*-/-*^ mice, which could facilitate their use in future pre-clinical drug trials.

## Materials and methods

### Animals

All experiments were approved by the Animal Experiment Committee (Dierexperimentencommissie) of the Leiden University Medical Center (protocol #13211) and executed following EU-guidelines. The *Sgca*^*-/-*^ (B6.129S6-Sgca^tm2Kcam^/J; α-sarcoglycan-deficient) mice [11] were kindly provided by Queensta Millet, University College London and the *Sgcd*^*-/-*^ (B6.129-Sgcd^tm1Mcn^/J; δ-sarcoglycan-deficient) mice [12] were obtained from Jackson Laboratory (Bar Harbor, ME, USA). Males were used for all experiments. Mice were bred in the Experimental Animal Facility of the Leiden University Medical Center. They were kept in ventilated cages at 20.5°C with 12 h of light/dark cycles and had *ad libitum* access to standard RM3 chow (SDS, Essex, UK) and water. Care was taken to limit the burden and distress for the animals as much as possible.

Twice monthly, *Sgca*^*-/-*^, *Sgcd*^*-/-*^ and C57BL/6J wild type male mice (n = 18 per genotype) were subjected to the four limb grip strength test and two and four limb hanging tests on consecutive days, from the age of 4 weeks to either 8, 16 or 24 weeks. At these ages, six males per genotype were sacrificed by cervical dislocation and muscles were dissected for analysis (see below) to allow direct comparisons between muscle function and pathology, while the remaining mice continued this functional test regime. The functional tests conducted were suitable and sensitive tests for muscle function in LGMD mice, based on our previous longitudinal study [10]. Standardized operating procedures from the TREAT-NMD network for *mdx* mice were implemented wherever possible [13].

### Four limb grip strength test

A grid attached to an isometric force transducer (Columbus Instruments, USA) was used to measure peak force of the fore and hind limbs. Hereto, the mouse was suspended, handled by the tail, above the grid which it naturally grasped with its four paws. The maximal force generated by the mouse to the grid when pulled away by the experimenter was recorded by the force transducer. This was repeated three times in a row after which the mouse got a short rest. The mouse was subjected to five series of three pulls, each followed by a resting period (15 pulls in total). The three highest values of the 15 pulls were averaged and normalized to body weight.

### Two limb hanging test

The mouse was suspended above a metal wire which was secured above a cage with bedding and released after it had grasped the wire with its fore limbs. The hanging time until fall was recorded. The mouse was allowed to use all four limbs and the tail during hanging if capable to do so. The test ended after a hanging time of 600 s was achieved or otherwise after three sessions. The maximum hanging time was utilized for analysis.

### Four limb hanging test

The mouse was placed on a grid, which was then turned upside down above a cage filled with bedding. The test ended when a hanging time of 600 s was achieved or, upon earlier fall, after three sessions. The maximum hanging time was utilised for analysis.

### Respiratory function analysis

Mice underwent respiratory function analysis with the whole-body plethysmograph (RM-80; Columbus Instruments, Columbus, OH, USA) [14] at the age of 8, 16 and 24 weeks. After 30 s acclimatization, the respiration signal was recorded for 120 s. The signal was digitized using a Minidigi digitizer and Axoscope 10 software (Axon Instruments/Molecular Devices, Sunnyvale, CA, USA) and analysed with the event detection feature of the Clampfit 10 program (Axon Instruments/Molecular Devices). This non-invasive monitoring system allowed for measurement of respiration rate and depth. The respiration amplitude was normalized to body weight.

### Muscle histology and morphology

Directly after sacrifice, the gastrocnemius, quadriceps, triceps, diaphragm and heart were isolated and snap frozen in liquid nitrogen cooled isopentane. Muscles were embedded in OCT compound and sections (8 μm thickness) were cut with the cryotome (Leica CM3050 S Research Cryostat; Amsterdam, the Netherlands) while intermediate sections were collected for RNA isolation. For all microscopical analyses pictures were taken from the entire cross-sectional area of a section originating from the middle part of the muscle at a ten times magnification with a BZ-X700 microscope (Keyence, Japan) and stitched with a BZ-X700 analyser version 1.3.0.3 (Keyence). Adobe Photoshop CC 2018 (Adobe Systems Corporation, San Jose, CA, United States) was used for background correction and ImageJ software (NIH) for analysis.

To assess overall pathology, slides were fixed in ice-cold acetone (Avantor; Arnhem, the Netherlands) for 5 min, stained with hematoxylin and eosin (H&E, Sigma-Aldrich, Zwijndrecht, the Netherlands) and mounted in Pertex mounting medium (VWR International B.V; Amsterdam, the Netherlands), according to conventional methods. Images were analysed by three to four examiners and the median of their assessments was used for calculations. Here, the amount of healthy versus unhealthy tissue (consisting of necrosis, fibrosis, inflammation and newly regenerated fibres) was assessed.

Sections were stained with Alizarin Red to quantify calcification area in muscle. Hereto, sections were fixed in ice-cold acetone (Avantor) for 10 min and exposed to Alizarin Red staining solution (Sigma-Aldrich) for 1 min. Then, sections were washed in acetone (Avantor) for 30 s and in 1:1 acetone/xylene for 15 s and finally incubated in xylene for 1.5 h and mounted in Pertex (VWR International B.V). The percentage of calcification was determined by two to three examiners by dividing Alizarin Red positive areas by the total area and the median of their assessments was used for final analysis.

For fibre size analyses, muscle sections were fixed with ice cold acetone (Avantor) and stained with a laminin primary antibody (ab11575, dilution 1:100 Abcam, USA) overnight at 4°C and with a goat-anti-rabbit Alexa 594 secondary antibody (A11012, dilution 1:1000, Life Technologies) for 1 h at room temperature. Sections were mounted with mounting medium containing DAPI (ProLong Antifade Reagents; Life Technologies). Per section five randomly chosen microscopic views were analysed resulting in an average total number of 1500-4000 fibres analysed per muscle. Fibres with an area <100 μm^2^ or >10000 μm^2^ were excluded from analysis.

Levels of collagen were quantified by Sirius Red staining. Sections were fixed in 4% paraformaldehyde for 10 min and 100% ethanol for 5 min. Thereafter, they were air dried for 30 min and rinsed in deionized water. Sections were stained with Sirius Red solution (Direct Red 80; Sigma-Aldrich) for 45 min, followed by washing with 0.5% acetic acid water for 5 min and rinsed in deionized water. Stepwise dehydration in ethanol (80%–90%–100%) was performed and after incubation in xylene for two times 5 min, sections were mounted in Pertex (VWR International B.V). The three to four examiners assessed the percentage of fibrosis by dividing the Sirius Red positive areas by the total area. When large variations in fibrosis levels occurred for a sample between evaluators, these samples were discussed and a compromise was achieved. The median of their results was used for analysis.

The percentage of fibrosis was measured for the heart. Hereto, sections were stained with collagen type I primary antibody (1310-01 Southern Biotech, dilution 1:100, Birmingham, USA) overnight at 4°C and with a donkey-anti-goat Alexa 488 secondary antibody (A11001, dilution 1:1000, Life Technologies) for 1 h at room temperature and mounted with mounting medium containing DAPI (ProLong Antifade Reagents; Life Technologies). Quantification of the collagen type I positive area and the total tissue area was performed by two examiners and the average of their acquired percentage of fibrosis was used for further analyses.

### RNA isolation and qPCR

Muscle sections were collected in 1.4 mm Zirconium Beads prefilled tubes (OPS Diagnostics, Lebanon, USA) and disrupted in TRIsure isolation reagent (GCBioetech, Alphen aan den Rijn, the Netherlands) using a MagNA Lyser (Roche Diagnostics). Total RNA was isolated and cleaned up by applying a NucleoSpin RNA II kit (Macherey-Nagel, Düren, Germany) according to the manufacturer’s instruction. cDNA was synthesized from 0.3-0.5 μg of total RNA (depending on the muscle type but kept constant between comparisons of different genotypes and ages of the same muscle) using random N6 primers (Thermo fisher scientific) and Bioscript enzyme (GCBiotech, Alphen aan den Rijn, the Netherlands) according to the manufacturer’s instructions. Gene expression levels were determined for cluster of differentiation 68 (*Cd68*), collagen type Iα1 (*Col1a1*), connective tissue growth factor (*Ctgf*), lectin, galactoside binding soluble 3 (*Lgals3*), lysyl oxidase (*Lox*), latent transforming growth factor beta binding protein 4 (*Ltbp4*), myosin heavy chain 3 (*Myh3*), myogenin (*MyoG*), NADPH oxidase 2 (*Nox2*), natriuretic peptide type A *(Nppa*), platelet derived growth factor receptor alpha polypeptide (*Pdgfrα*), peroxisome proliferator-activated receptor γ (*Pparγ*), sarcoplasmic reticulum Ca^2+^ ATPase2α (*Serca2α*), signal transducer and activator of transcription 3 (*Stat3*) and vascular endothelial growth factor (*Vegf*) in triplo by quantitative PCR, using SensiMix reagents (GCBiotech) and the LightCycler 480 (Roche), using a program consisting of 45 cycles of 95°C (10 s), 58°C (30 s) and 72°C (20 s). The LinReg qPCR method was used to analyse gene expression levels and values were normalized utilizing the housekeeping gene *Hmbs*. Primer sequences and gene functions are provided in S1 Table.

#### miRNA analysis

Mice were anaesthetized with isoflurane and blood was collected in an Eppendorf tube after orbital bleeding, after which mice were sacrificed by cervical dislocation. Clotting was allowed for 10 min at room temperature, and samples were subsequently stored on ice. Serum was obtained after 10 min spinning at 13.000 rcf and stored until further processing at -80°C. Serum samples of 8- and 16-weeks-old mice were used for analysis. Due to limited availability of serum from 24-weeks-old mice, serum samples of 34 weeks old C57BL/6J wild type, *Sgca*^*-/-*^ and *Sgcd*^*-/-*^ males (n = 5–6 per group) of our previous natural history study [10] were included in further analyses. The set-up of this study was comparable to the previous one and samples had been obtained in the same manner. Total RNA from 100 μL serum was isolated in 500 μL TRIsure (Bioline, London, United Kingdom). Hereto, 3 μL of three synthetic *Caenorhabditis elegans* miRNAs (*cel-miR-39-3p*, *cel-miR-54-3p* and *cel-miR-238-3p*) (Eurogentec, Maastricht, the Netherlands) with a concentration of 5 nM was added. cDNA synthesis was performed using 47.25 ng of total RNA. First, microRNAs (miRNAs) were polyA tailed using poly(A) polymerase (ABMgood, Richmond, BC, Canada) and oligo(dT) adapter (GAATCGAGCACCAGTTACGCATGCCGAGGTCGACTTCCTAGATTTTTTTTTTTTTTTTTTTTTTTTT), whereupon cDNA was generated using EasyScript RTase (ABMgood). cDNA was diluted five times and quantitative PCR was performed in triplo using FastStart PCR Buffer containing 20 mM MgCl_2_ (Roche), nucleotide mix, PCR grade (Roche), EvaGreen Dye (Biotium, Hayward, CA, United States) and FastStart Taq DNA polymerase (Roche) on the LightCycler 480 (Roche), using a program consisting of 50 cycles of 95°C (10 s), 60°C (30 s) and 72°C (20 s). Specific forward primers for *cel-miR-39-3p*, *cel-miR-54-3p*, *cel-miR-238-3*p, *mmu-miR-1a-3p*, *mmu-miR-23a-3p*, *mmu-miR-30e-3p*, *mmu-miR-133a-3p*, *mmu-miR-133b-3p*, *mmu-miR-148a-3p*, *mmu-miR-206-3p*, *mmu-miR-434-3p* and *mmu-miR-451a-3p* in combination with an universal reverse primer complementary to the adapter sequence of the RT-primer were used. Calculations of relative expression were done with Lin-RegPCR quantitative PCR data analysis software V11.3. Average expression of the spike-ins *cel-miR-39-3p*, *cel-miR-54-3p* and *cel-miR-238-3p* was used for data normalisation to correct for differences in starting concentration. Samples that gave inconsistent results for one or more of the three spike-ins were left out of all analyses, since normalisation was not possible. In addition, a sample of a particular miRNA of interest was left out of analysis in case of inconsistent or missing values to ensure reliability of the results. The absence of haemolysis was confirmed by the ratio of miR-451a: miR-23a. All sera had a value of <5 (S2A and S2B Fig) [15]. Primer sequences and miRNA functions are provided in S2 Table.

### Statistical analyses

For statistical analyses, R Studio version 1.1.453 [16] and Prism 7 (GraphPad Software Inc., La Jolla, CA, USA) were used. Values are presented as means ± standard deviation (SD). For all muscle function assessments, linear mixed-effects model analysis was performed using fixed effects for genotype and age (including their interaction), and per mouse a random intercept and a random age-effect. Since grip strength did not show a linear pattern over time, a quadratic effect of age was also included as a fixed and random effect. Genotypes were compared with an ANOVA test using a likelihood ratio test. Histology, gene expression, miR levels, and heart—body weight ratio were analysed with a two-way ANOVA between genotypes and age groups with Tukey’s multiple comparison test to correct for multiple testing. To assess difference in fibre size distribution, the distribution of fibres was summarised to a single value per mouse by calculating the proportion of small fibres, where small was defined as <1500 μm^2^ for gastrocnemius, quadriceps and triceps and <500 μm^2^ for diaphragm. A two-way ANOVA test was subsequently used between genotypes and age groups, followed by Tukey’s post hoc correction for multiple comparisons. Statistical significance was set at *p<0*.*05*. All results of histological, gene expression and miRNA analyses are provided in S3 Table.

## Results

### Muscle function

**Fig 1:**
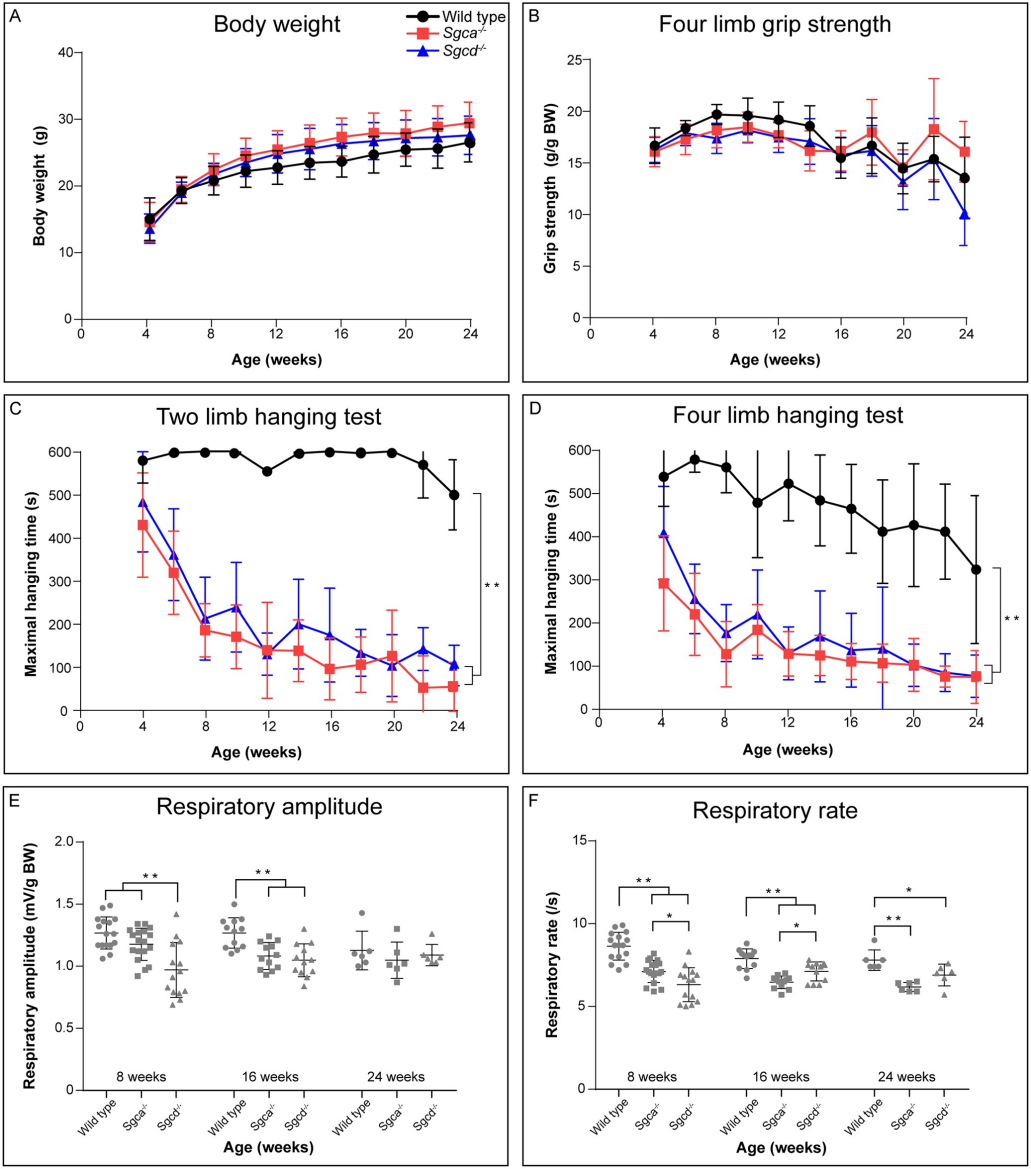
Functional performance and respiratory function of wild type, *Sgca*^*-/-*^ and *Sgcd*^*-/-*^ males. (A) Body weight. (B) Four limb grip strength normalised for body weight. (C) Maximal two limb hanging times. (D) Maximal four limb hanging times. (E) Respiratory amplitude corrected for body weight. (F) Respiratory rate. N = 6–18 male mice per group. Values are presented as mean ± SD. *p<0.05, **p<0.01.

*In vivo* muscle function of *Sgca*^*-/-*^ and *Sgcd*^*-/-*^ mice was assessed on a bi-weekly basis for a period of either 4, 12 or 20 weeks, starting at the age of four weeks. Functional test types were chosen based on previous experiments [10]. Functionality was compared to C57BL/6J wild type mice and only males were included as their muscle performance is more impaired than that of females in the *Sgcd*^*-/-*^ strain [10]. Body weight did not differ between the genotypes (Fig 1A). No difference in muscle strength was observed between the genotypes as assessed by the four limb grip strength test (Fig 1B). To examine muscle function and fatigability two and four limb hanging tests were performed (Fig 1C and 1D). In both tests wild type mice greatly outperformed *Sgca*^*-/-*^ and *Sgcd*^*-/-*^ mice (*p*<0.01), of which the muscle function declined with age. No differences were observed between *Sgca*^*-/-*^ and *Sgcd*^*-/-*^ mice.

Since respiratory dysfunction has been observed in LGMD patients and animal models [10, 17], whole-body plethysmography was performed. Respiratory amplitude, normalised to body weight, was lower in *Sgca*^*-/-*^ and/or *Sgcd*^*-/-*^ mice versus wild type mice at 8 and 16 weeks of age, but not at 24 weeks (Fig 1E). Respiratory rate was decreased in both LGMD models compared to wild type mice at all ages, and differed between the *Sgca*^*-/-*^ and *Sgcd*^*-/-*^ mice at 8 and 16, but not at 24 weeks of age (Fig 1F). Respiratory function did not decline with age in any of the genotypes.

### Muscle quality

**Fig 2:**
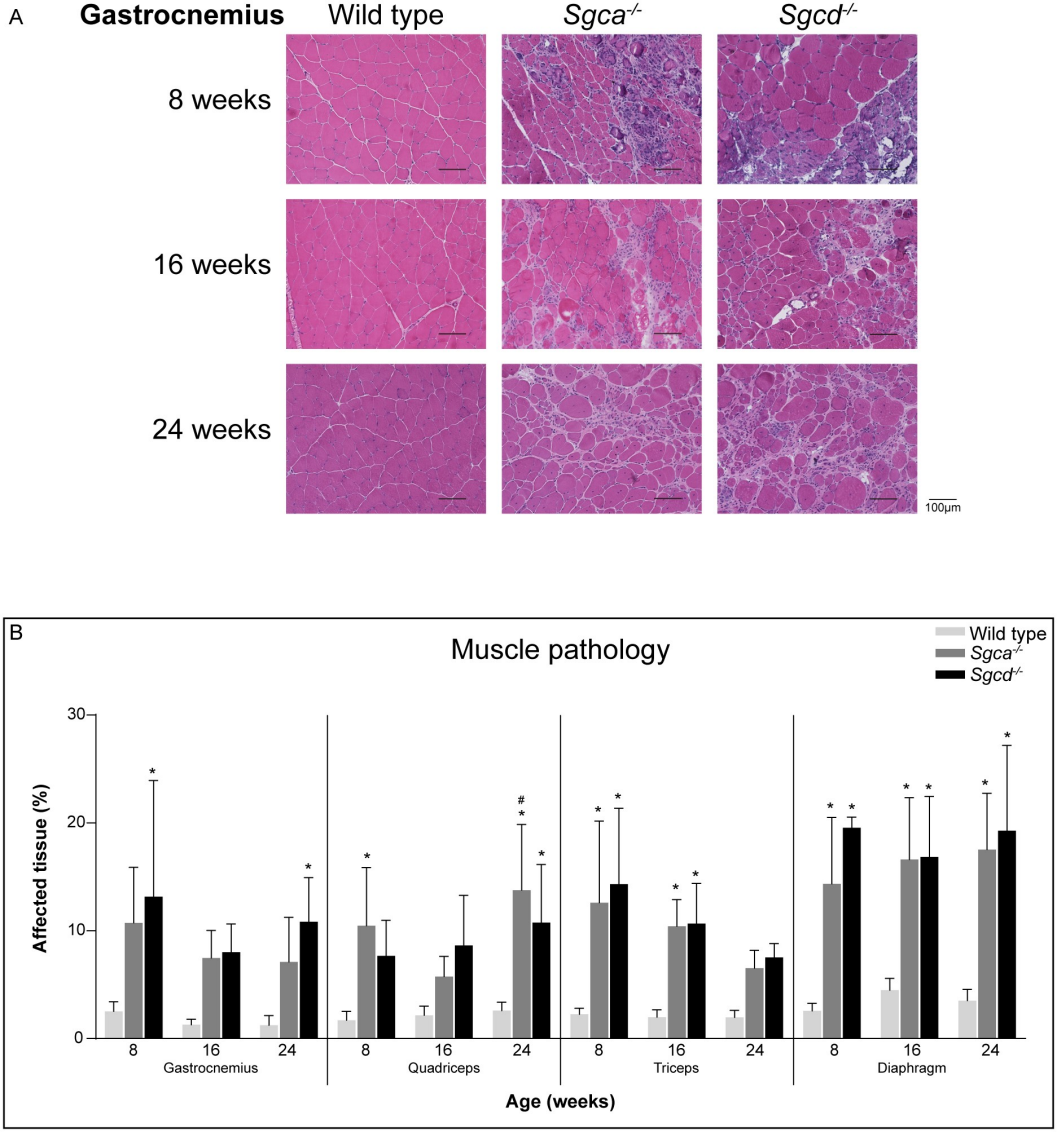
Histological analysis of muscle quality. (A) Representative images (20x magnification) of H&E staining of the gastrocnemius of each genotype and age group. Scale bars represent 100 μm. (B) Quantification of affected tissue. N = 6 male mice per group. *p<0.05 vs age-matched wild type mice, #*p*<0.05 over time (same genotype).

Overall muscle pathology (consisting of necrosis, fibrosis, inflammation and newly regenerated fibres) was assessed by H&E staining for the gastrocnemius, quadriceps, triceps and diaphragm (Fig 2 and S1 Fig). Already at 8 weeks of age, muscle quality was compromised in both *Sgca*^*-/-*^ and *Sgcd*^*-/-*^ compared to wild type mice for all examined muscles. Pathology did not differ between the *Sgca*^*-/-*^ and *Sgcd*^*-/-*^ models, and remained stable within the investigated age period for all muscles. The diaphragm was more severely affected than the limb muscles, which is in line with previous observations in sarcoglycanopathy and other dystrophic mouse models [10, 18, 19].

### Fibrosis and fatty infiltration

**Fig 3:**
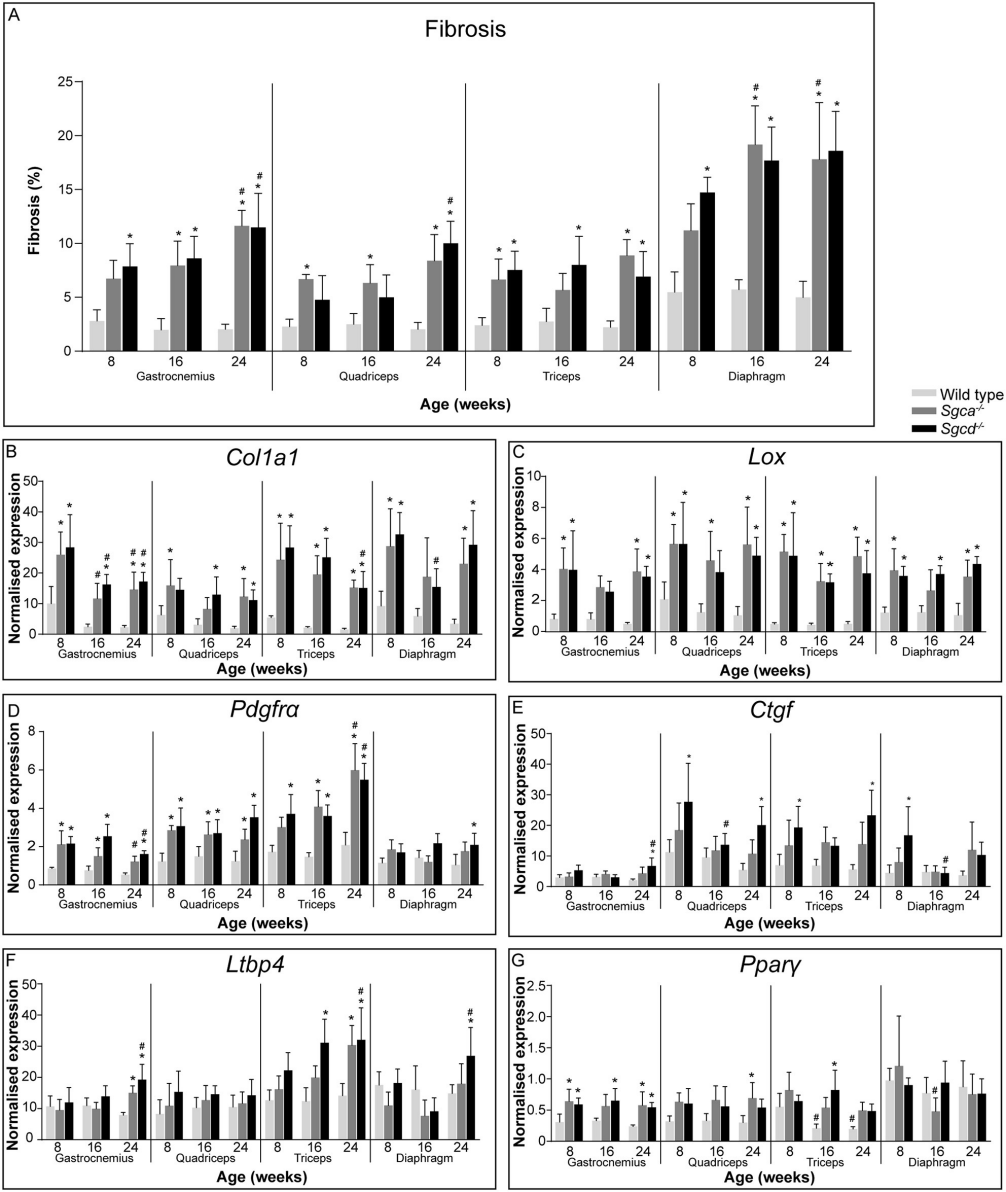
Fibrosis and fat formation in *Sgca*^*-/-*^ and *Sgcd*^*-/-*^ males. (A) Quantification of fibrosis by Sirius Red staining. (B-F) Gene expression of fibrotic markers *Col1a1* (B), *Lox* (C), *Pdgfrα* (D), *Ctgf* (E) and *Ltbp4* (F). (G) Gene expression of *Pparγ*, a marker for fatty infiltration. N = 5-6 male mice per group. *p<0.05 vs age-matched wild type mice, #*p*<0.05 over time (same genotype).

During disease progression, degeneration of muscle fibres causes large infiltrates of inflammatory cells and eventually replacement of myofibrils by fibrotic and adipose tissue [11, 20]. To examine fibrosis, muscle sections were stained with Sirius Red (Fig 3A). Already from 8 weeks of age onwards, large areas of fibrosis were seen in all analysed muscles of both LGMD strains. Fibrotic lesions increased in abundance during ageing for some, but not all, muscles. In correspondence with the H&E staining quantification, the diaphragm contained the largest proportion of fibrosis.

Furthermore, gene expression levels of markers for fibrosis were measured (*i*.*e*. *Col1a1*, *Lox*, *Pdgfrα*, *Ctgf* and *Ltbp4*). Out of these genes, the expression of *Col1a1* (Fig 3B), *Lox* (Fig 3C) and *Pdgfrα* (Fig 3D) was most drastically upregulated in *Sgca*^*-/-*^ and *Sgcd*^*-/-*^ mice, already from early age onwards. For *Col1a1*, a small decrease with age was seen in the gastrocnemius and triceps (Fig 3B). *Pdgfrα* levels were upregulated in all muscles except for the diaphragm of *Sgca*^*-/-*^ and *Sgcd*^*-/-*^ mice. Furthermore, in the gastrocnemius of both LGMD models a small decrease over time was seen, whereas levels were increased in the triceps (Fig 3D). More moderate changes were observed for *Ctgf* (Fig 3E) and *Ltbp4* expression (Fig 3F). *Ctgf* expression was increased in *Sgcd*^*-/-*^, but not in *Sgca*^-/-^ males. *Ltbp4* expression solely increased at 24 months, mainly in *Sgcd*^*-/-*^ males (Fig 3F).

Expression of *Pparγ*, a regulator of adipocyte differentiation, was assessed as a marker for fatty infiltration. Increased *Pparγ* expression was seen in both LGMD mouse strains, most prominently in the gastrocnemius. In the diaphragm, however, *Pparγ* expression did not differ between wild type and dystrophic mice (Fig 3G).

### Regeneration

**Fig 4:**
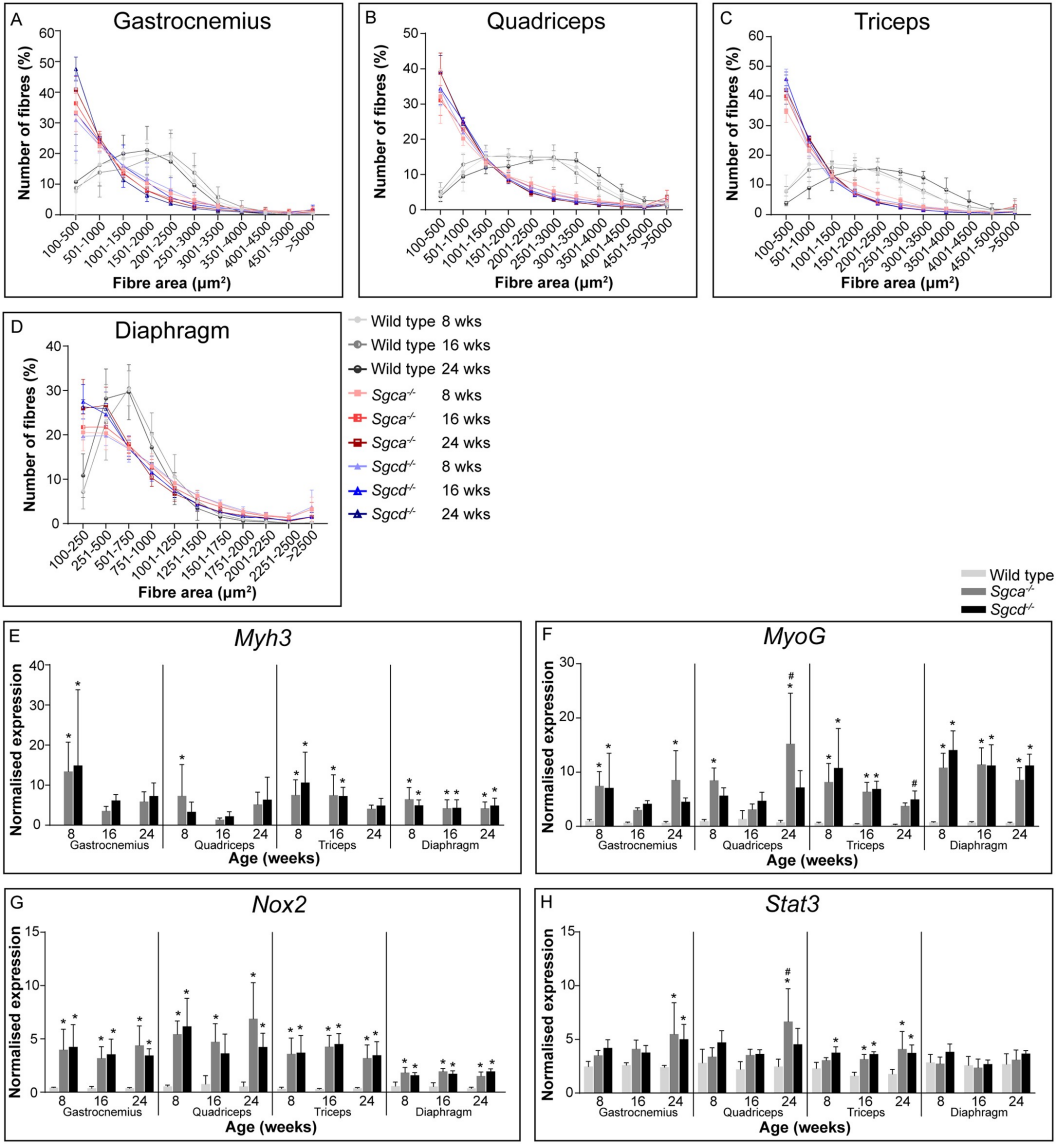
Regeneration in *Sgca*^*-/-*^ and *Sgcd*^*-/-*^ males. (A-D) Muscle fibre area distribution measured by staining with laminin. (E-H) Gene expression of *Myh3* (E). Since *Myh3* was not detectable in wild type mice, values were put at zero. *MyoG* (F), *Nox2* (G) and *Stat3* (H). N = 5-6 male mice per group. *p<0.05 vs age-matched wild type mice, #*p*<0.05 over time (same genotype).

Cycles of degeneration and regeneration are a hallmark of muscular dystrophies. Fibre size distribution was determined utilising a laminin staining to segment individual fibres (Fig 4A–4D). A shift towards smaller fibre sizes, an indicator of regeneration, was seen in all examined muscles of both LGMD models. At all ages, most fibres were smaller than 1000 μm^2^ for gastrocnemius, quadriceps and triceps for *Sgca*^*-/-*^ and *Sgcd*^*-/-*^ mice, whereas in wild type mice the majority was 1000-3000 μm^2^ (Fig 4A–4C). In the diaphragm, smaller fibre sizes were observed in general, also for wild type mice, compared to the other muscles. Even though a shift towards smaller fibres was seen in *Sgca*^*-/-*^ and *Sgcd*^*-/-*^ mice, this was less prominent than for other muscles (Fig 4D). No large changes over time were observed in the examined muscles, neither did fibre size differ between the two LGMD models.

Expression levels of several regeneration markers (*Myh3*, *MyoG*, *Nox2* and *Stat3*) were assessed (Fig 4E–4G). The expression of most genes was upregulated in the LGMD models from 8 weeks of age onwards and few changes over time were observed. *Myh3*, expressed by recently regenerated fibres, was nearly undetectable in wild type mice. In *Sgca*^*-/-*^ and *Sgcd*^*-/-*^ mice, however, significantly higher levels were found in all analysed muscles at most time points (Fig 4E). *MyoG*, a marker of early regeneration, was also upregulated in LGMD mice (Fig 4F). *Nox2*, a marker of regeneration and inflammation, was upregulated in all muscles of *Sgca*^*-/-*^ and *Sgcd*^*-/-*^ mice regardless of their age (Fig 4G). Less abundant changes were seen for *Stat3*. Expression was only statistically significantly increased in triceps of both dystrophic models at all time points (except for *Sgca*^*-/-*^ mice at 8 weeks of age), and in gastrocnemius and quadriceps of 24 weeks old mice *Sgca*^*-/-*^ and/or *Sgcd*^*-/*^ mice (Fig 4H). For *Sgca*^*-/-*^ mice also a small increase over time was observed in quadriceps.

### Inflammation and calcification

**Fig 5:**
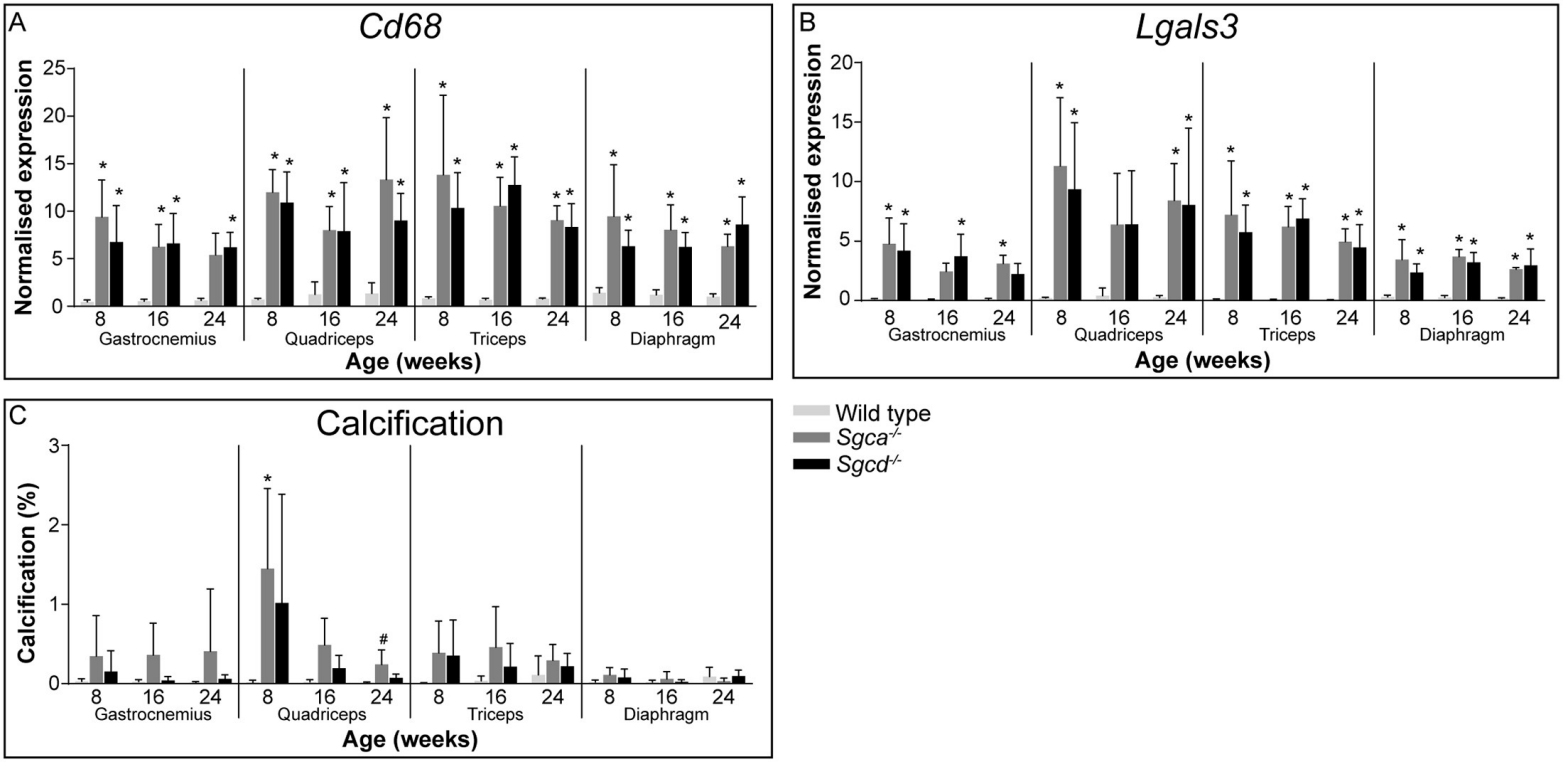
Inflammation and calcification in *Sgca*^*-/-*^ and *Sgcd*^*-/-*^ males. (A and B) Gene expression of *Cd68* (A) and *Lgals3* (B). (C) Quantification of calcification by the Alizarin Red staining. N = 5-6 male mice per group. *p<0.05 vs age-matched wild type mice. #*p*<0.05 over time (same genotype).

Another hallmark of dystrophic muscle is the infiltration of inflammatory cells. To measure the extent of inflammation, expression levels of *Cd68* and *Lgals3* were determined (Fig 5A and 5B). Expression of both markers was upregulated in all examined muscles of the *Sgca*^*-/-*^ and *Sgcd*^*-/-*^ mice at almost all timepoints. Similarly to findings for fibrosis and regeneration, no differences during ageing were observed.

To assess whether calcification is involved in the pathology of *Sgca*^*-/-*^ and *Sgcd*^*-/-*^ mice, muscle sections were stained with Alizarin Red. Whereas no calcified fibres were observed in wild type mice, skeletal muscles of both sarcoglycanopathy mouse models contained small groups of calcified fibres, regardless of the animal’s age (Fig 5C). The extent differed between individuals.

### miRNA

**Fig 6:**
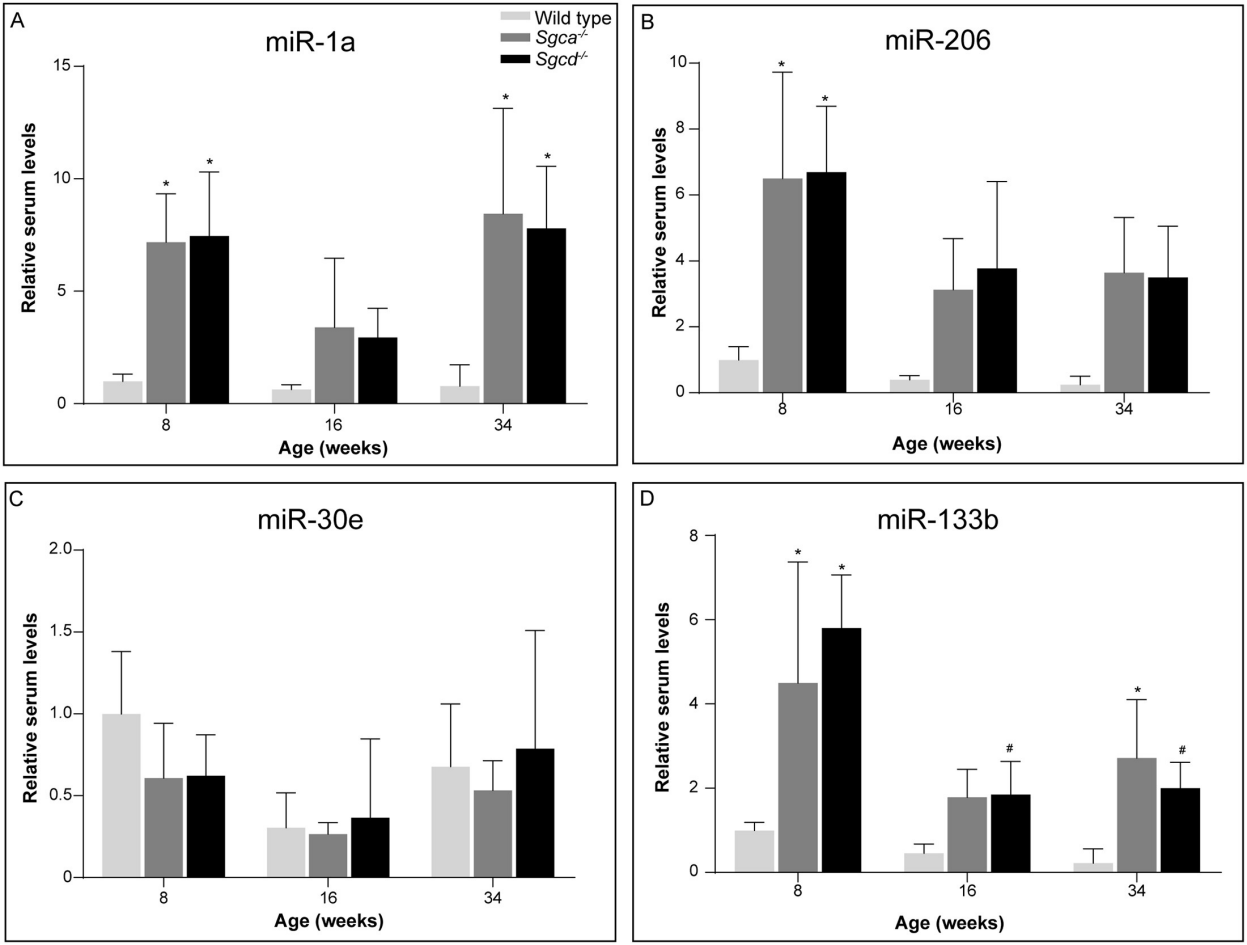
miRNA serum levels. (A-D) Relative levels of miR-1a (A), miR-206 (B), miR-30e (C) and miR-133b (D) in 8, 16 and 34 weeks old mice. N = 4-6 male mice per group. For miR-30e, the level of 16-weeks-old wild type mice is based on n = 3. Levels of 8-weeks-old wild type mice were put at 1. *p<0.05 vs age-matched wild type mice, #*p*<0.05 over time (same genotype).

miRNAs regulate mRNA expression by post-transcriptional cleavage or repression [21, 22]. They show tissue-specific expression patterns and play a role in several pathophysiological conditions, among which muscular dystrophies [23, 24]. Upon muscle damage miRNAs can leak into the blood stream. Therefore, we looked in serum at the levels of several miRNAs, involved in myogenic proliferation and/or differentiation [23, 25–29] (for more information on specific roles see S2 Table). Some of these are specifically expressed in muscle (myomiRs) (Fig 6 and S2 Fig). With limited sample available for the 24 weeks old mice, we used serum of 34 weeks old *Sgca*^*-/-*^, *Sgcd*^*-/-*^ and C57BL/6J males instead, collected in our previous natural history study [10]. Mainly in young *Sgca*^*-/-*^ and *Sgcd*^*-/-*^ mice, increased levels of miR-1a and miR-206, mi-RNAs which promote myogenic differentiation and are abundant in muscle, were observed (Fig 6A and 6B). However, no differences were observed for miR-30e (Fig 6C) and miR-148a (S2C Fig). Increased levels of miR-133a (S2D Fig) and miR-133b (Fig 6D) are indicative for active myoblast proliferation in *Sgca*^*-/-*^ and *Sgcd*^*-/-*^ males. A small decrease in expression during ageing was observed in all genotypes, although this was only significant in *Sgcd*^*-/-*^ mice. miR-434 has been shown to inhibit apoptosis and is downregulated in mice during ageing [30]. No differences were observed between the LGMD models and the wild types (S2E Fig).

### Cardiac involvement

**Fig 7:**
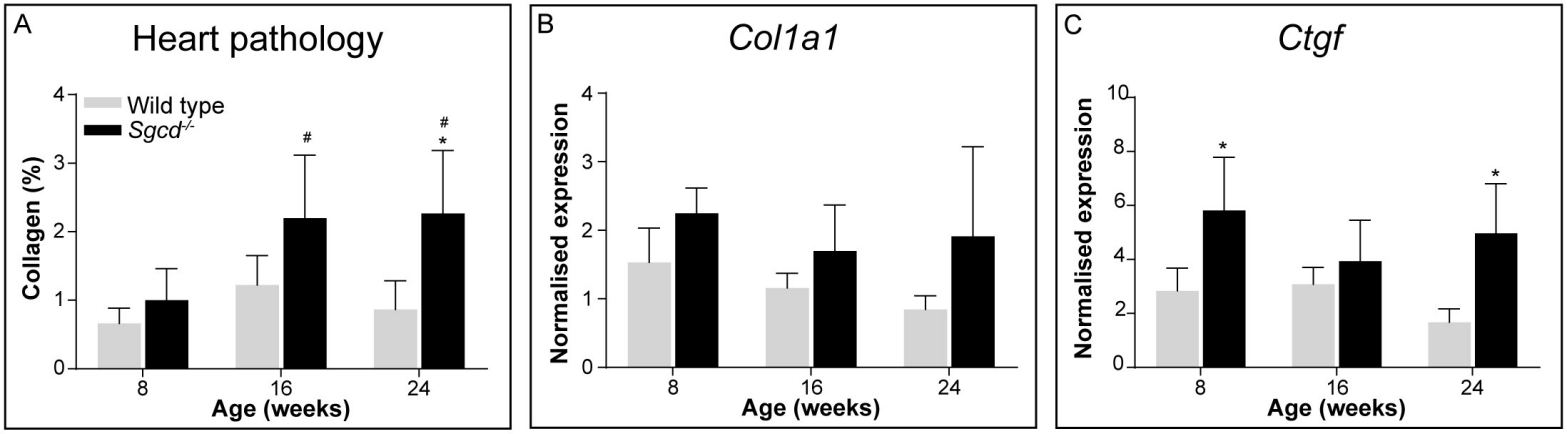
Heart pathology in *Sgcd*^*-/-*^ males. (A) Collagen type I staining of fibrotic area. Gene expression of *Col1a1* (B) and *Ctgf* (C). N = 6 male mice per group. *p<0.05 vs age-matched wild type mice, #*p*<0.05 over time (same genotype).

Cardiomyopathy has been observed in *Sgcd*^*-/-*^ mice [9, 10] and LGMD2F patients [8, 31], but not in *Sgca*^*-/-*^ mice [10]. Therefore, heart pathology was only assessed in *Sgcd*^*-/-*^ mice. To assess cardiac hypertrophy, the heart to body weight ratio was determined. No differences with wild type mice were observed at any age (S3A Fig). Furthermore, the amount of collagen (fibrotic tissue) was analysed (Fig 7A). Whereas in young *Sgcd*^*-/-*^ mice no cardiac fibrosis was observed yet, from the age of 16 weeks onwards some fibrotic areas were present in the dystrophic mice. A modest increase in expression of fibrotic genes (*Col1a1* and *Ctgf*) was also observed, mainly for *Ctgf* (Fig 7B and 7C). The increased fibrosis in the *Sgcd*^*-/-*^ hearts was not accompanied by an altered expression of genes involved in cardiac function (*Serca2a* [32, 33], *Nppa* [34] and *Vegf* [35]) (S3B–S3D Fig).

## Discussion

At the moment, specific therapies for the sarcoglycanopathies LGMD2D and 2F are lacking and patients are treated according to the care guidelines [36, 37]. For LGMD2D clinical research into gene therapy is ongoing, but for LGMD2F therapeutic development is still only in pre-clinical stages [38, 39]. Animal models are important tools for understanding disease mechanisms and pre-clinical research on potential therapies. Although description of their natural course of the disease is vital for these studies, full characterization of the *Sgca*^*-/-*^ and *Sgcd*^*-/-*^ mice is lacking. In a previous study we assessed the disease pathology in adult mice (at 34 weeks of age), which showed a severe phenotype [10]. To determine if disease features are already present at younger ages and how they develop over time, we here investigated several pathological hallmarks in 8-, 16- and 24-weeks-old mice.

*In vivo* muscle function was already impaired in the youngest mice investigated (8 weeks of age) and showed a rapid decline with age. These results are in line with those obtained during the previous natural history study [10]. Especially in the hanging tests large differences with wild type mice were observed, similar to those seen in mouse models for Duchenne muscular dystrophy (DMD) [13, 40]. Respiratory dysfunction is common in sarcoglycanopathies [1, 17, 31] and was indeed already seen in both models at young age. However, in contrast to human patients, no further decline was seen during ageing. While in the previous study respiratory amplitude of 15-weeks-old *Sgca*^*-/-*^ and *Sgcd*^*-/-*^ mice only showed a small tendency towards decline [10], in the current study this decrease was statistically significant at 16 weeks of age. Results obtained for the respiratory rate do correspond between both studies.

Histological analyses showed that all examined muscles, especially the diaphragm, were severely affected in both LGMD models. Fibrotic, inflammatory and necrotic tissues were prominent. Furthermore, muscles were characterized by small calibre fibres which contained centrally located nuclei, indicative of muscle regeneration in the LGMD models at all ages. Especially in skeletal muscles, occasional groups of calcified fibres were present in both LGMD models. This was however not as extensive as that seen in the D2-*mdx* (a mouse model for DMD) and β-sarcoglycan deficient mice [41–43]. Already at eight weeks of age muscles were severely affected and remained relatively stable over time. It is likely that disease pathology starts at early age. It would therefore be interesting to investigate neonatal mice in future studies.

Several secondary signalling pathways are also disturbed due to the absence of sarcoglycans. These are, amongst others, pathways involved in fibrosis, adiposis, inflammation and regeneration. We here showed that several genes involved were upregulated in both LGMD models. Most markers already showed differential expression at eight weeks of age and remained relatively stable over time. In addition to gene expression, also miRNAs are valuable biomarkers for disease mechanisms. Especially myomiRs involved in myoblast proliferation and differentiation (*e*.*g*. by affecting MyoG and/or MyoD signalling), such as miR-1a, miR-133a/b and miR-206 [23, 28], were increased in the bloodstream of *Sgca*^*-/-*^ and *Sgcd*^*-/-*^ mice at most ages, suggestive of ongoing regeneration and damage. This confirms an early study showing an increase of these miRNAs in *Sgca*^-/-^ mice [44]. To our knowledge miRNA serum levels have not been examined specifically in LGMD2D patients or LGMD2F patients/mouse models. Similar changes, however, have been observed in other muscular dystrophies, such as other types of LGMD (LGMD2A, 2B and 2C) and DMD [24, 44–46]. In one study on the miR profile of LGMD2A/B patients, contrasting effects were found (a decrease in muscle miR levels rather than an increase) [47]. It could be that the rate of regeneration differs between LGMD types 2A/B and types 2D/F. Another explanation may be that in that study muscle tissue was used, whereas in our, and most other, studies, serum samples were used. Increased miR serum levels can only been observed if these miRs also leak out of the muscle tissue into the bloodstream. Therefore, it would be interesting to investigate miR expression levels in muscle tissue of *Sgca*^*-/-*^ and *Sgcd*^*-/-*^ mice.

Cardiac involvement is common in LGMD2F patients, but not in LGMD2D patients [17, 48]. This is because δ-sarcoglycan, but not α-sarcoglycan, is also expressed in cardiomyocytes and arterial smooth muscle cells. Its absence leads to myofiber damage and causes disturbances in the vascular function, leading to cardiomyopathy [9, 49]. We indeed confirmed previously that only *Sgcd*^*-/-*^ mice suffer from cardiomyopathy [10], and here showed that fibrosis in the heart could be detected from 16 weeks of age onwards. This was accompanied by elevated expression of fibrotic markers in the heart, but not by altered expression of genes related to heart function. More in depth analysis of cardiac function of *Sgcd*^*-/-*^ mice would be of interest. Future studies should focus in greater detail on the involvement of cardiomyopathy in these mice.

Overall the results indicate that *Sgca*^*-/-*^ and *Sgcd*^*-/-*^ mice resemble the muscular dystrophy features observed in patients, with an early onset of pathology [2, 36]. This allows utilization of these models for the testing of therapeutic interventions from an early age onwards. Especially examination of effects on the diaphragm is important, since this is the most severely affected muscle in both models and most likely also in patients. Further studies are needed to establish at which age pathology is starting in these strains. An important difference between LGMD2D and 2F patients and the mouse models is that the disease progression is limited in mice. While muscle function steadily declined, muscle pathology did not drastically increase in severity with age. This could have several reasons. Firstly, mice are known for their relatively good muscle regeneration capacity, which was also observed in these mice. In *mdx* mice cycles of de-and regeneration occur between 3 to 8 weeks of age, whereupon stabilisation of the disease is seen due to the regeneration and low levels of necrosis [50, 51]. This might also be the case for *Sgca*^*-/-*^ and *Sgcd*^*-/-*^ mice. Secondly, the relative length of the growth and maturation period is much shorter in mice compared to humans. Thirdly, the load on the muscles is reduced in mice due to their small body size and, since mice walk on four limbs and humans on two, different muscles are stressed [52].

In conclusion, in addition to our previous study of 34-weeks-old mice, this cross-sectional study further underscores that *Sgca*^*-/-*^ and *Sgcd*^*-/-*^ mice are representative models for LGMD2D and LGMD2F, respectively. Therefore they are useful for pre-clinical testing of therapeutic interventions.

## Supporting information

S1 Fig Histological analysis of muscle quality. Representative images (20x magnification) of H&E staining of the gastrocnemius of each genotype and age group. Scale bars represent 100 μm.
(TIF)

S2 Fig miRNA serum levels. (A-E) Levels of miR-23a (A), miR-451a (B), miR-148a (C), miR-133a (D) and miR-434 (E) in 8, 16 and 34 weeks old males. N = 3-6 male mice per group, except for miR-133a in 8-weeks old *Sgca*^*-/-*^ mice (n = 2). *p<0.05 vs age-matched wild type mice, #*p*<0.05 over time (same genotype).
(TIF)

S3 Fig Heart pathology of *Sgcd*^*-/-*^ mice. (A) Heart to body weight ratio. (B-D) Expression of genes related to heart function *Serca2α* (B), *Nppa* (C) and *Vegf* (D). N = 6 male mice per group.
(TIF)

S1 Table Primer sequences and functions of genes used for gene expression analysis. (DOCX)

S2 Table Primer sequences used for miRNA analysis. (DOCX)

S3 Table Statistical analysis of histology, gene expression and serum miRNA levels. NA = not applicable; ND = not determined; ns = not significant. *p*-values after correcting for multiple testing.
(XLSX)

## References

[1] Murphy AP, Straub V. The Classification, Natural History and Treatment of the Limb Girdle Muscular Dystrophies. Journal of neuromuscular diseases. 2015;2(s2):S7–S19. 10.3233/JND-150105 27858764 PMC5271430

[2] Nigro V, Savarese M. Genetic basis of limb-girdle muscular dystrophies: the 2014 update. Acta myologica: myopathies and cardiomyopathies: official journal of the Mediterranean Society of Myology. 2014;33(1):1–12. 24843229 PMC4021627

[3] Durbeej M, Campbell KP. Muscular dystrophies involving the dystrophin-glycoprotein complex: an overview of current mouse models. Current opinion in genetics & development. 2002;12(3):349–61. 12076680 10.1016/s0959-437x(02)00309-x

[4] Straub V, Campbell KP. Muscular dystrophies and the dystrophin-glycoprotein complex. Current opinion in neurology. 1997;10(2):168–75. 9146999 10.1097/00019052-199704000-00016

[5] Cohn RD, Campbell KP. Molecular basis of muscular dystrophies. Muscle & nerve. 2000;23(10):1456–71. 11003781 10.1002/1097-4598(200010)23:10<1456::aid-mus2>3.0.co;2-t

[6] Campbell KP. Three muscular dystrophies: loss of cytoskeleton-extracellular matrix linkage. Cell. 1995;80(5):675–9. 10.1016/0092-8674(95)90344-5 7889563

[7] Thompson R, Straub V. Limb-girdle muscular dystrophies—international collaborations for translational research. Nature reviews Neurology. 2016;12(5):294–309. 10.1038/nrneurol.2016.35 27033376

[8] Blain AM, Straub VW. delta-Sarcoglycan-deficient muscular dystrophy: from discovery to therapeutic approaches. Skeletal muscle. 2011;1(1):13 10.1186/2044-5040-1-13 21798091 PMC3156636

[9] Coral-Vazquez R, Cohn RD, Moore SA, Hill JA, Weiss RM, Davisson RL, et al Disruption of the sarcoglycan-sarcospan complex in vascular smooth muscle: a novel mechanism for cardiomyopathy and muscular dystrophy. Cell. 1999;98(4):465–74. 10.1016/s0092-8674(00)81975-3 10481911

[10] Pasteuning-Vuhman S, Putker K, Tanganyika-de Winter CL, Boertje-van der Meulen JW, van Vliet L, Overzier M, et al Natural disease history of mouse models for limb girdle muscular dystrophy types 2D and 2F. PloS one. 2017;12(8):e0182704 10.1371/journal.pone.0182704 28797108 PMC5552258

[11] Duclos F, Straub V, Moore SA, Venzke DP, Hrstka RF, Crosbie RH, et al Progressive muscular dystrophy in alpha-sarcoglycan-deficient mice. The Journal of cell biology. 1998;142(6):1461–71. 10.1083/jcb.142.6.1461 9744877 PMC2141773

[12] Hack AA, Lam MY, Cordier L, Shoturma DI, Ly CT, Hadhazy MA, et al Differential requirement for individual sarcoglycans and dystrophin in the assembly and function of the dystrophin-glycoprotein complex. Journal of cell science. 2000;113 (Pt 14):2535–44. 10862711 10.1242/jcs.113.14.2535

[13] Aartsma-Rus A, van Putten M. Assessing functional performance in the mdx mouse model. Journal of visualized experiments: JoVE. 2014(85). 10.3791/51303 PMC4158772 24747372

[14] van der Pijl EM, van Putten M, Niks EH, Verschuuren JJGM, Aartsma-Rus A, Plomp JJ, et al Characterization of neuromuscular synapse function abnormalities in multiple Duchenne muscular dystrophy mouse models. European Journal of Neuroscience. 2016;43(12):1623–35. 10.1111/ejn.13249 27037492

[15] Blondal T, Jensby Nielsen S, Baker A, Andreasen D, Mouritzen P, Wrang Teilum M, et al Assessing sample and miRNA profile quality in serum and plasma or other biofluids. Methods (San Diego, Calif). 2013;59(1):S1–6. 10.1016/j.ymeth.2012.09.015 23036329

[16] RStudio Team. RStudio: Integrated Development for R. 1.1.453 ed Boston, MA: RStudio Inc; 2016.

[17] Fayssoil A, Ogna A, Chaffaut C, Chevret S, Guimaraes-Costa R, Leturcq F, et al Natural History of Cardiac and Respiratory Involvement, Prognosis and Predictive Factors for Long-Term Survival in Adult Patients with Limb Girdle Muscular Dystrophies Type 2C and 2D. PloS one. 2016;11(4):e0153095 10.1371/journal.pone.0153095 27120200 PMC4847860

[18] Jakubiec-Puka A, Biral D, Krawczyk K, Betto R. Ultrastructure of diaphragm from dystrophic alpha-sarcoglycan-null mice. Acta biochimica Polonica. 2005;52(2):453–60. 15990925

[19] van Putten M, Kumar D, Hulsker M, Hoogaars WM, Plomp JJ, van Opstal A, et al Comparison of skeletal muscle pathology and motor function of dystrophin and utrophin deficient mouse strains. Neuromuscular disorders: NMD. 2012;22(5):406–17. 10.1016/j.nmd.2011.10.011 22284942

[20] Turk R, Sterrenburg E, van der Wees CG, de Meijer EJ, de Menezes RX, Groh S, et al Common pathological mechanisms in mouse models for muscular dystrophies. FASEB journal: official publication of the Federation of American Societies for Experimental Biology. 2006;20(1):127–9. 16306063 10.1096/fj.05-4678fje

[21] Bartel DP. MicroRNAs: genomics, biogenesis, mechanism, and function. Cell. 2004;116(2):281–97. 10.1016/s0092-8674(04)00045-5 14744438

[22] Bartel DP. MicroRNAs: target recognition and regulatory functions. Cell. 2009;136(2):215–33. 10.1016/j.cell.2009.01.002 19167326 PMC3794896

[23] Eisenberg I, Alexander MS, Kunkel LM. miRNAS in normal and diseased skeletal muscle. Journal of cellular and molecular medicine. 2009;13(1):2–11. 10.1111/j.1582-4934.2008.00524.x 19175696 PMC3072056

[24] Eisenberg I, Eran A, Nishino I, Moggio M, Lamperti C, Amato AA, et al Distinctive patterns of microRNA expression in primary muscular disorders. Proceedings of the National Academy of Sciences of the United States of America. 2007;104(43):17016–21. 10.1073/pnas.0708115104 17942673 PMC2040449

[25] Chen JF, Mandel EM, Thomson JM, Wu Q, Callis TE, Hammond SM, et al The role of microRNA-1 and microRNA-133 in skeletal muscle proliferation and differentiation. Nature genetics. 2006;38(2):228–33. 10.1038/ng1725 16380711 PMC2538576

[26] Guess MG, Barthel KK, Harrison BC, Leinwand LA. miR-30 family microRNAs regulate myogenic differentiation and provide negative feedback on the microRNA pathway. PloS one. 2015;10(2):e0118229 10.1371/journal.pone.0118229 25689854 PMC4331529

[27] Kim HK, Lee YS, Sivaprasad U, Malhotra A, Dutta A. Muscle-specific microRNA miR-206 promotes muscle differentiation. The Journal of cell biology. 2006;174(5):677–87. 10.1083/jcb.200603008 16923828 PMC2064311

[28] Koutsoulidou A, Mastroyiannopoulos NP, Furling D, Uney JB, Phylactou LA. Expression of miR-1, miR-133a, miR-133b and miR-206 increases during development of human skeletal muscle. BMC developmental biology. 2011;11:34 10.1186/1471-213X-11-34 21645416 PMC3132729

[29] Zhang J, Ying ZZ, Tang ZL, Long LQ, Li K. MicroRNA-148a promotes myogenic differentiation by targeting the ROCK1 gene. The Journal of biological chemistry. 2012;287(25):21093–101. 10.1074/jbc.M111.330381 22547064 PMC3375532

[30] Pardo PS, Hajira A, Boriek AM, Mohamed JS. MicroRNA-434-3p regulates age-related apoptosis through eIF5A1 in the skeletal muscle. Aging. 2017;9(3):1012–29. 10.18632/aging.101207 28331100 PMC5391215

[31] Politano L, Nigro V, Passamano L, Petretta V, Comi LI, Papparella S, et al Evaluation of cardiac and respiratory involvement in sarcoglycanopathies. Neuromuscular Disorders. 2001;11(2):178–85. 11257475 10.1016/s0960-8966(00)00174-7

[32] Frank KF, Bolck B, Erdmann E, Schwinger RH. Sarcoplasmic reticulum Ca2+-ATPase modulates cardiac contraction and relaxation. Cardiovascular research. 2003;57(1):20–7. 10.1016/s0008-6363(02)00694-6 12504810

[33] Goonasekera SA, Lam CK, Millay DP, Sargent MA, Hajjar RJ, Kranias EG, et al Mitigation of muscular dystrophy in mice by SERCA overexpression in skeletal muscle. The Journal of clinical investigation. 2011;121(3):1044–52. 10.1172/JCI43844 21285509 PMC3049367

[34] Potter LR, Yoder AR, Flora DR, Antos LK, Dickey DM. Natriuretic peptides: their structures, receptors, physiologic functions and therapeutic applications. Handbook of experimental pharmacology. 2009(191):341–66. 10.1007/978-3-540-68964-5_15 19089336 PMC4855512

[35] Klagsbrun M, D’Amore PA. Vascular endothelial growth factor and its receptors. Cytokine & growth factor reviews. 1996;7(3):259–70. 8971481 10.1016/s1359-6101(96)00027-5

[36] Bushby K. Diagnosis and management of the limb girdle muscular dystrophies. Practical neurology. 2009;9(6):314–23. 10.1136/jnnp.2009.193938 19923111

[37] Norwood F, de Visser M, Eymard B, Lochmuller H, Bushby K, Force EGT. EFNS guideline on diagnosis and management of limb girdle muscular dystrophies. European journal of neurology. 2007;14(12):1305–12. 10.1111/j.1468-1331.2007.01979.x 18028188

[38] Mendell JR, Rodino-Klapac LR, Rosales-Quintero X, Kota J, Coley BD, Galloway G, et al Limb-girdle muscular dystrophy type 2D gene therapy restores alpha-sarcoglycan and associated proteins. Annals of neurology. 2009;66(3):290–7. 10.1002/ana.21732 19798725 PMC6014624

[39] Straub V, Bertoli M. Where do we stand in trial readiness for autosomal recessive limb girdle muscular dystrophies? Neuromuscular disorders: NMD. 2016;26(2):111–25. 10.1016/j.nmd.2015.11.012 26810373

[40] van Putten M, de Winter C, van Roon-Mom W, van Ommen GJ, t Hoen PA, Aartsma-Rus A. A 3 months mild functional test regime does not affect disease parameters in young mdx mice. Neuromuscular disorders: NMD. 2010;20(4):273–80. 10.1016/j.nmd.2010.02.004 20307983

[41] Giovannelli G, Giacomazzi G, Grosemans H, Sampaolesi M. Morphological and functional analyses of skeletal muscles from an immunodeficient animal model of limb-girdle muscular dystrophy type 2E. Muscle & nerve. 2018. 10.1002/mus.26112 PMC6099247 29476695

[42] Coley WD, Bogdanik L, Vila MC, Yu Q, Van Der Meulen JH, Rayavarapu S, et al Effect of genetic background on the dystrophic phenotype in mdx mice. Human molecular genetics. 2016;25(1):130–45. 10.1093/hmg/ddv460 26566673 PMC4690497

[43] van Putten M, Putker K, Overzier M, Adamzek WA, Pasteuning-Vuhman S, Plomp JJ, et al Natural disease history of the D2 -mdx mouse model for Duchenne muscular dystrophy. FASEB journal: official publication of the Federation of American Societies for Experimental Biology. 2019:fj201802488R. 10.1096/fj.201802488R PMC6593893 30933664

[44] Vignier N, Amor F, Fogel P, Duvallet A, Poupiot J, Charrier S, et al Distinctive serum miRNA profile in mouse models of striated muscular pathologies. PloS one. 2013;8(2):e55281 10.1371/journal.pone.0055281 23418438 PMC3572119

[45] Li X, Li Y, Zhao L, Zhang D, Yao X, Zhang H, et al Circulating Muscle-specific miRNAs in Duchenne Muscular Dystrophy Patients. Molecular therapy Nucleic acids. 2014;3:e177 10.1038/mtna.2014.29 25050825 PMC4121518

[46] Zaharieva IT, Calissano M, Scoto M, Preston M, Cirak S, Feng L, et al Dystromirs as serum biomarkers for monitoring the disease severity in Duchenne muscular Dystrophy. PloS one. 2013;8(11):e80263 10.1371/journal.pone.0080263 24282529 PMC3840009

[47] Aguennouz M, Lo Giudice C, Licata N, Rodolico C, Musumeci O, Fanin M, et al MicroRNA signatures predict dysregulated vitamin D receptor and calcium pathways status in limb girdle muscle dystrophies (LGMD) 2A/2B. Cell biochemistry and function. 2016;34(6):414–22. 10.1002/cbf.3202 27558075

[48] Schade van Westrum SM, Dekker LR, de Voogt WG, Wilde AA, Ginjaar IB, de Visser M, et al Cardiac involvement in Dutch patients with sarcoglycanopathy: a cross-sectional cohort and follow-up study. Muscle & nerve. 2014;50(6):909–13. 24619517 10.1002/mus.24233

[49] Goehringer C, Rutschow D, Bauer R, Schinkel S, Weichenhan D, Bekeredjian R, et al Prevention of cardiomyopathy in delta-sarcoglycan knockout mice after systemic transfer of targeted adeno-associated viral vectors. Cardiovascular research. 2009;82(3):404–10. 10.1093/cvr/cvp061 19218289

[50] Willmann R, Possekel S, Dubach-Powell J, Meier T, Ruegg MA. Mammalian animal models for Duchenne muscular dystrophy. Neuromuscular disorders: NMD. 2009;19(4):241–9. 10.1016/j.nmd.2008.11.015 19217290

[51] Radley-Crabb HG, Marini JC, Sosa HA, Castillo LI, Grounds MD, Fiorotto ML. Dystropathology Increases Energy Expenditure and Protein Turnover in the Mdx Mouse Model of Duchenne Muscular Dystrophy. PloS one. 2014;9(2):e89277 10.1371/journal.pone.0089277 24586653 PMC3929705

[52] Grounds MD, Radley HG, Lynch GS, Nagaraju K, De Luca A. Towards developing standard operating procedures for pre-clinical testing in the mdx mouse model of Duchenne muscular dystrophy. Neurobiology of disease. 2008;31(1):1–19. 10.1016/j.nbd.2008.03.008 18499465 PMC2518169

