# Supplementary figures and images for: Cross-sectional study into age-related pathology of mouse models for limb girdle muscular dystrophy types 2D and 2F
Source: PLoS One. 2019 Aug 20;14(8):e0220665. doi: 10.1371/journal.pone.0220665 (PMC6701749; doi:10.1371/journal.pone.0220665)

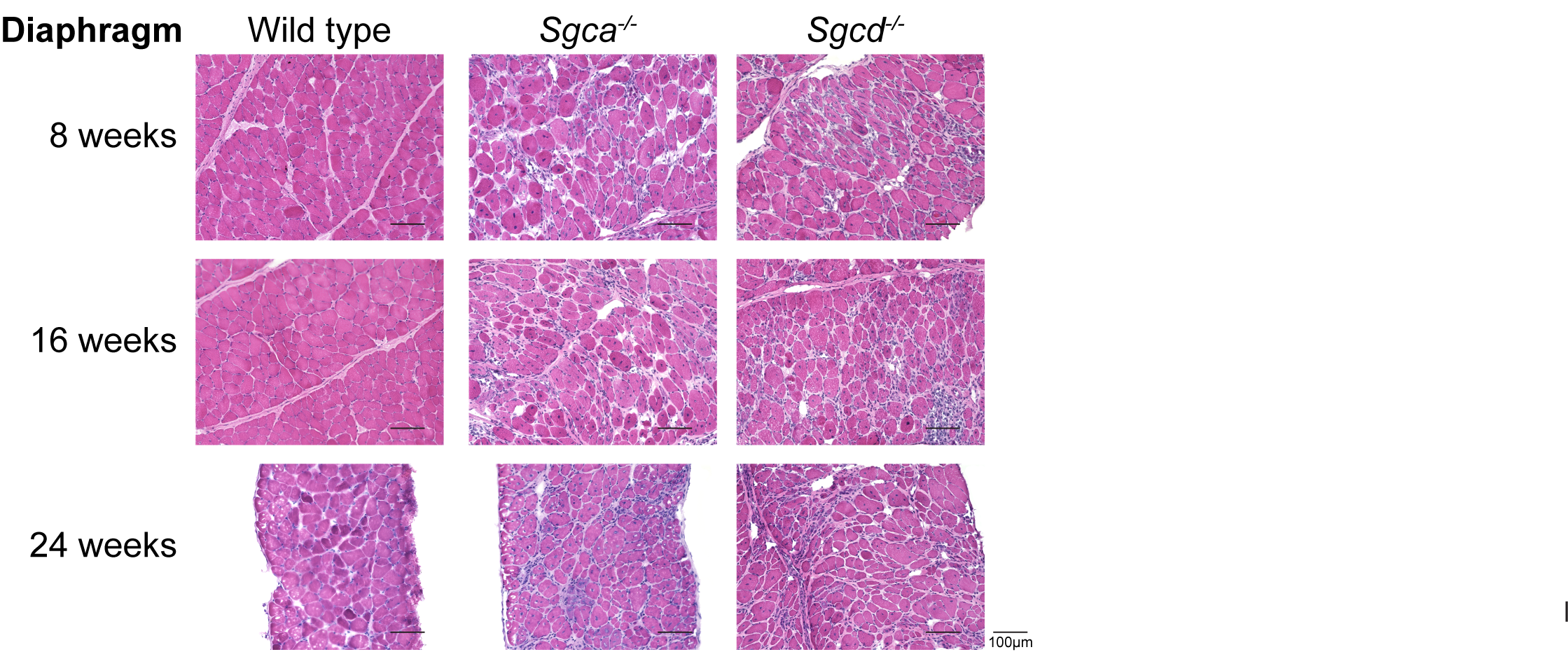

Supplement: S1 Fig — Representative images (20x magnification) of H&E staining of the gastrocnemius of each genotype and age group. Scale bars represent 100 μm. (TIF) [file pone.0220665.s001.tif]

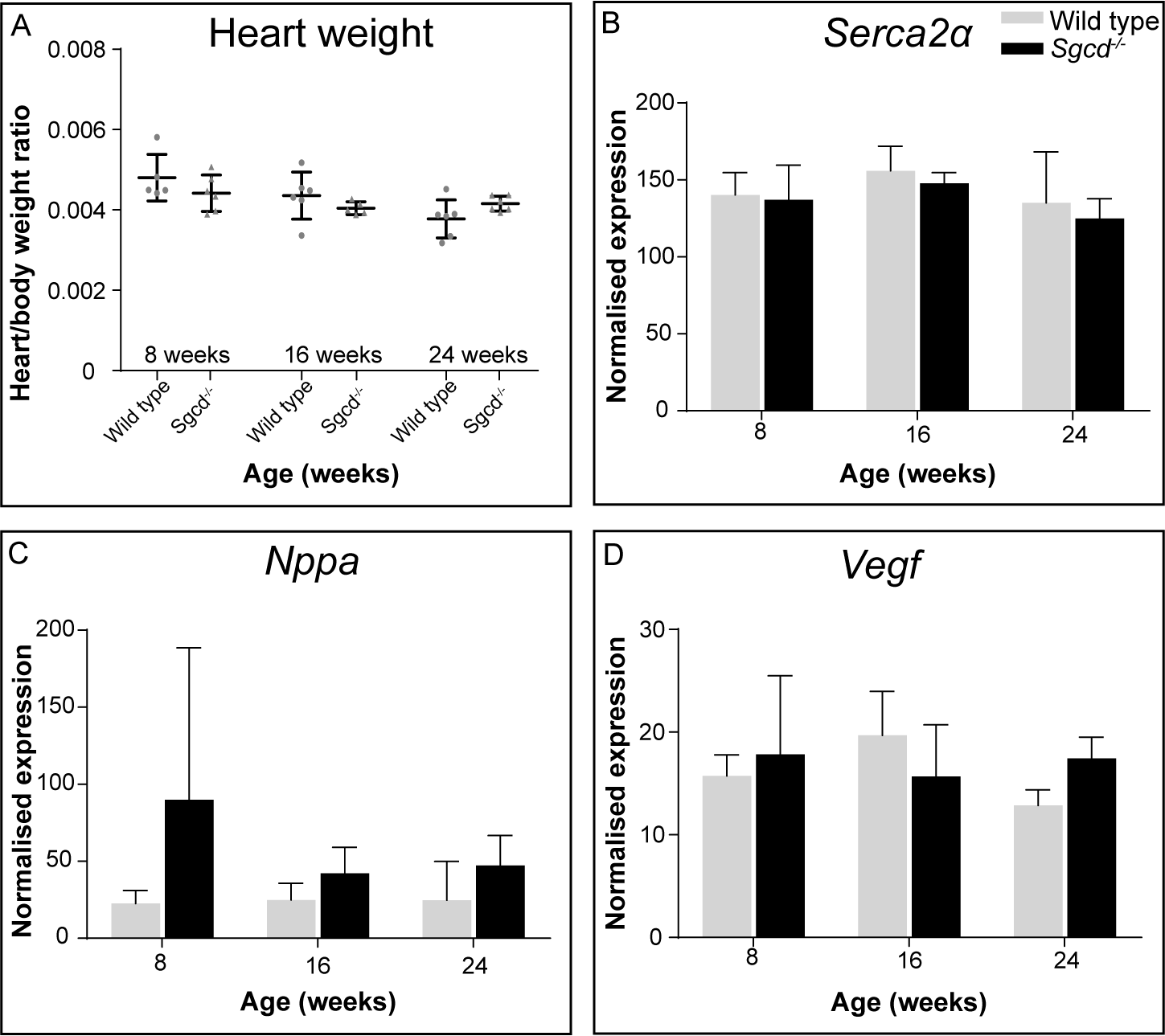

Supplement: S3 Fig — (A) Heart to body weight ratio. (B-D) Expression of genes related to heart function Serca2α (B), Nppa (C) and Vegf (D). N = 6 male mice per group. (TIF) [file pone.0220665.s003.tif]
