# Supplementary material for: Cross-sectional study into age-related pathology of mouse models for limb girdle muscular dystrophy types 2D and 2F
Source: PLoS One. 2019 Aug 20;14(8):e0220665. doi: 10.1371/journal.pone.0220665 (PMC6701749; doi:10.1371/journal.pone.0220665)
Supplement: S1 Table — (DOCX) [file pone.0220665.s004.docx]

**S1 Table. Primer sequences used for gene expression analysis**

| **Gene** | **Full name** | **Primer** | **Sequence (5' - 3')** | **Function** |
| --- | --- | --- | --- | --- |
| ***Hmbs*** | Hydroxymethylbilane synthase | forward | TCCCTGAAGGATGTGCCTAC | Housekeeping gene |
|  |  | reverse | AAGGGTTTTCCCGTTTGC |  |
| ***Cd68*** | Cluster of differentiation 68 | forward | CTTCGGGCCATGTTT**C**TCT | Macrophage marker (1) |
|  |  | reverse | AGAGGGGCTGGTAGGTTGAT |  |
| ***Col1a1*** | Collagen, type I, alpha 1 | forward | ATGTTCAGCTTTGTGGACCT | Extracellular matrix component synthesizes by fibroblasts (2) |
|  |  | reverse | CAGCTGACTTCAGGGATGT |  |
| ***Ctgf*** | Connective tissue growth factor | forward | AGCTGGGAGAACTGTGTACG | Proliferation of fibroblasts (3) |
|  |  | reverse | GCCAAATGTGTCTTCCAGTC |  |
| ***Lgals3*** | Lectin, galactoside binding soluble 3 | forward | CAACCATCGGATGAAGAACC | Interaction of immune cells with the extracellular matrix (4) |
|  |  | reverse | TTCCCACTCCTAAGGCACAC |  |
| ***Lox*** | Lysyl oxidase | forward | CAGAGGAGAGTGGCTGAAGG | Cross-linking of collagen and elastin and is essential for development of cardiovascular and respiratory systems, and development of skin and connective tissue (5, 6) |
|  |  | reverse | CTGCCGCATAGGTGTCATAA |  |
| ***Ltbp4*** | Latent transforming growth factor beta binding protein 4 | forward | TTTAGAGAACTGCGCGGAAG | Regulates the release of *Tgf-β* from the extracellular matrix (7-9) |
|  |  | reverse | GCCACTCACTTGGTTGGAGT |  |
| ***Myh3*** | Embryonic myosin heavy chain | forward | CGCAGAATCGCAAGTCAATA | Developmental marker, actively regeneration fibres (10) |
|  |  | reverse | CAGGAGGTCTTGCTCACTCC |  |
| ***Myog*** | Myogenin | forward | CCCAACCCAGGAGATCATTT | Early regeneration marker (required for myotube formation) (11) |
|  |  | reverse | GTCTGGGAAGGCAACAGACA |  |
| ***Nox2*** | NADPH oxidase 2 | forward | ACCTTACTGGCTGGGATGAA | Induced by reactive oxygen species (12) |
|  |  | reverse | TCCTCATCATGGTGCACAG |  |
| ***Nppa*** | Natriuretic peptide type A | forward | CTGCAACAGCTTCCGGTACC | Control of extracellular fluid volume and electrolyte homeostasis (13) |
|  |  | reverse | GCTGCGTGACACACCACAAG |  |
| ***Pdgfrα*** | Platelet-derived growth factor receptor α | forward | GATAGTGGAGAACCTGTTGC | Muscle regeneration and fibrosis (14) |
|  |  | reverse | TCAGTCTCTGTTCGTCCAGG |  |
| ***Pparγ*** | Peroxisome proliferator-activated receptor gamma | forward | GACCAGGGAGTTCCTCAAAA | Regulator of adipocyte differentiation (15) |
|  |  | reverse | CAGGTTGTCTTGGATGTCCTC |  |
| ***Serca2α*** | Sarcoplasmic reticulum Ca^2+^ ATPase2α | forward | ACCTGGAACAACCCGCAATAC | Maintenance of calcium homeostasis (16, 17) |
|  |  | reverse | CCCAACCTCAGTCATGCAGAG |  |
| ***Stat3*** | Signal transducer and activator of transcription 3 | forward | GCTGCTGCATCTTCTGTCTG | Activated by extracellular signals such as cytokines and growth factors, inducing gene transcription (18) |
|  |  | reverse | TGAAGGTGGTGGAGAACCTC |  |
| ***Vegf*** | Vascular endothelial growth factor | forward | CAGGCTGCTGTAACGATGAA | Regulator of endothelial cell proliferation and angiogenesis, induced in ischemic conditions (19) |
|  |  | reverse | GCATTCACATCTGCTGTGCT |  |

**References**

1. Aartsma-Rus, A., and Spitali, P. (2015) Circulating Biomarkers for Duchenne Muscular Dystrophy. *Journal of neuromuscular diseases* **2**, S49-S58

2. Rossert, J., Terraz, C., and Dupont, S. (2000) Regulation of type I collagen genes expression. *Nephrology, dialysis, transplantation : official publication of the European Dialysis and Transplant Association - European Renal Association* **15 Suppl 6**, 66-68

3. Ramazani, Y., Knops, N., Elmonem, M. A., Nguyen, T. Q., Arcolino, F. O., van den Heuvel, L., Levtchenko, E., Kuypers, D., and Goldschmeding, R. (2018) Connective tissue growth factor (CTGF) from basics to clinics. *Matrix biology : journal of the International Society for Matrix Biology* **68-69**, 44-66

4. t Hoen, P. A., van der Wees, C. G., Aartsma-Rus, A., Turk, R., Goyenvalle, A., Danos, O., Garcia, L., van Ommen, G. J., den Dunnen, J. T., and van Deutekom, J. C. (2006) Gene expression profiling to monitor therapeutic and adverse effects of antisense therapies for Duchenne muscular dystrophy. *Pharmacogenomics* **7**, 281-297

5. Spurney, C. F., Knoblach, S., Pistilli, E. E., Nagaraju, K., Martin, G. R., and Hoffman, E. P. (2008) Dystrophin-deficient cardiomyopathy in mouse: expression of Nox4 and Lox are associated with fibrosis and altered functional parameters in the heart. *Neuromuscular disorders : NMD* **18**, 371-381

6. Lucero, H. A., and Kagan, H. M. (2006) Lysyl oxidase: an oxidative enzyme and effector of cell function. *Cellular and molecular life sciences : CMLS* **63**, 2304-2316

7. Heydemann, A., Ceco, E., Lim, J. E., Hadhazy, M., Ryder, P., Moran, J. L., Beier, D. R., Palmer, A. A., and McNally, E. M. (2009) Latent TGF-beta-binding protein 4 modifies muscular dystrophy in mice. *The Journal of clinical investigation* **119**, 3703-3712

8. Flanigan, K. M., Ceco, E., Lamar, K. M., Kaminoh, Y., Dunn, D. M., Mendell, J. R., King, W. M., Pestronk, A., Florence, J. M., Mathews, K. D., Finkel, R. S., Swoboda, K. J., Gappmaier, E., Howard, M. T., Day, J. W., McDonald, C., McNally, E. M., Weiss, R. B., and United Dystrophinopathy, P. (2013) LTBP4 genotype predicts age of ambulatory loss in Duchenne muscular dystrophy. *Annals of neurology* **73**, 481-488

9. van den Bergen, J. C., Hiller, M., Böhringer, S., Vijfhuizen, L., Ginjaar, H. B., Chaouch, A., Bushby, K., Straub, V., Scoto, M., Cirak, S., Humbertclaude, V., Claustres, M., Scotton, C., Passarelli, C., Lochmüller, H., Muntoni, F., Tuffery-Giraud, S., Ferlini, A., Aartsma-Rus, A. M., Verschuuren, J. J. G. M., t Hoen, P. A. C., and Spitali, P. (2015) Validation of genetic modifiers for Duchenne muscular dystrophy: a multicentre study assessingSPP1andLTBP4variants. *Journal of Neurology, Neurosurgery & Psychiatry* **86**, 1060-1065

10. Saad, A. D., Obinata, T., and Fischman, D. A. (1987) Immunochemical Analysis of Protein Isoforms in Thick Myofilaments of Regenerating Skeletal-Muscle. *Developmental biology* **119**, 336-349

11. Venuti, J. M., Morris, J. H., Vivian, J. L., Olson, E. N., and Klein, W. H. (1995) Myogenin is required for late but not early aspects of myogenesis during mouse development. *The Journal of cell biology* **128**, 563-576

12. Shin, J., Tajrishi, M. M., Ogura, Y., and Kumar, A. (2013) Wasting mechanisms in muscular dystrophy. *The international journal of biochemistry & cell biology* **45**, 2266-2279

13. Potter, L. R., Yoder, A. R., Flora, D. R., Antos, L. K., and Dickey, D. M. (2009) Natriuretic peptides: their structures, receptors, physiologic functions and therapeutic applications. *Handbook of experimental pharmacology*, 341-366

14. Mueller, A. A., van Velthoven, C. T., Fukumoto, K. D., Cheung, T. H., and Rando, T. A. (2016) Intronic polyadenylation of PDGFRalpha in resident stem cells attenuates muscle fibrosis. *Nature* **540**, 276-279

15. Rosen, E. D., and MacDougald, O. A. (2006) Adipocyte differentiation from the inside out. *Nature reviews. Molecular cell biology* **7**, 885-896

16. Frank, K. F., Bolck, B., Erdmann, E., and Schwinger, R. H. (2003) Sarcoplasmic reticulum Ca2+-ATPase modulates cardiac contraction and relaxation. *Cardiovascular research* **57**, 20-27

17. Goonasekera, S. A., Lam, C. K., Millay, D. P., Sargent, M. A., Hajjar, R. J., Kranias, E. G., and Molkentin, J. D. (2011) Mitigation of muscular dystrophy in mice by SERCA overexpression in skeletal muscle. *The Journal of clinical investigation* **121**, 1044-1052

18. Levy, D. E., and Darnell, J. E., Jr. (2002) Stats: transcriptional control and biological impact. *Nature reviews. Molecular cell biology* **3**, 651-662

19. Klagsbrun, M., and D'Amore, P. A. (1996) Vascular endothelial growth factor and its receptors. *Cytokine & growth factor reviews* **7**, 259-270
