# Supplementary material for: Cross-sectional study into age-related pathology of mouse models for limb girdle muscular dystrophy types 2D and 2F
Source: PLoS One. 2019 Aug 20;14(8):e0220665. doi: 10.1371/journal.pone.0220665 (PMC6701749; doi:10.1371/journal.pone.0220665)
Supplement: S2 Table — (DOCX) [file pone.0220665.s005.docx]

**S2 Table. Primer sequences used for miRNA expression analysis**

| **miRNA** | **Sequence (5' - 3')** | **Function** |
| --- | --- | --- |
| Universal reverse primer | GAATCGAGCACCAGTTACGC |  |
| cel-miR-39-3p | UCACCGGGUGUAAAUCAGCUUG | Spike-in control miRNA |
| cel-miR-54-3p | UACCCGUAAUCUUCAUAAUCCGAG | Spike-in control miRNA |
| cel-miR-238-3p | UUUGUACUCCGAUGCCAUUCAGA | Spike-in control miRNA |
| 39 | GTCACCGGGTGTAAATCAG | Normalisation control |
| 54 | CAGTACCCGTAATCTTCATAATCC | Normalisation control |
| 238 | AGTTTGTACTCCGATGCCA | Normalisation control |
| 1a | CGCAGTGGAATGTAAAGAAGTATG | Promote myogenesis and myogenic differentiation (1, 2); upregulated by myogenin and MyoD (3, 4) |
| 23a | AGATCACATTGCCAGGGA | Quality control (absence of haemolysis) (5, 6) |
| 30e | GCAGCTTTCAGTCGGATGT | Promote myogenic differentiation (7) |
| 133a | TGGTCCCCTTCAACCAG | Promote myogenic proliferation (1); expression regulated by regulated by myogenin, MyoD, Mef2, SRF(4, 8) |
| 133b | CAGTTTGGTCCCCTTCAAC | Promote myogenic proliferation and differentiation (1, 3); upegulated by myogenin and MyoD (3, 4) |
| 148a | AGTCAGTGCACTACAGAACT | Promote myogenic differentiation (9) |
| 206 | GCAGTGGAATGTAAGGAAGTGT | Promote myogenic differentiation (10); upregulated by MyoD (3, 11) |
| 434 | AGTTTGAACCATCACTCGAC | Inhibit apoptosis (12) |
| 451a | GCAGAAACCGTTACCATTACTG | Quality control (absence of haemolysis) (5, 6) |

**References**

1. Chen, J. F., Mandel, E. M., Thomson, J. M., Wu, Q., Callis, T. E., Hammond, S. M., Conlon, F. L., and Wang, D. Z. (2006) The role of microRNA-1 and microRNA-133 in skeletal muscle proliferation and differentiation. *Nature genetics* **38**, 228-233

2. Eisenberg, I., Alexander, M. S., and Kunkel, L. M. (2009) miRNAS in normal and diseased skeletal muscle. *Journal of cellular and molecular medicine* **13**, 2-11

3. Koutsoulidou, A., Mastroyiannopoulos, N. P., Furling, D., Uney, J. B., and Phylactou, L. A. (2011) Expression of miR-1, miR-133a, miR-133b and miR-206 increases during development of human skeletal muscle. *BMC developmental biology* **11**, 34

4. Rao, P. K., Kumar, R. M., Farkhondeh, M., Baskerville, S., and Lodish, H. F. (2006) Myogenic factors that regulate expression of muscle-specific microRNAs. *Proceedings of the National Academy of Sciences of the United States of America* **103**, 8721-8726

5. Blondal, T., Jensby Nielsen, S., Baker, A., Andreasen, D., Mouritzen, P., Wrang Teilum, M., and Dahlsveen, I. K. (2013) Assessing sample and miRNA profile quality in serum and plasma or other biofluids. *Methods (San Diego, Calif.)* **59**, S1-6

6. Shah, J. S., Soon, P. S., and Marsh, D. J. (2016) Comparison of Methodologies to Detect Low Levels of Hemolysis in Serum for Accurate Assessment of Serum microRNAs. *PloS one* **11**, e0153200

7. Guess, M. G., Barthel, K. K., Harrison, B. C., and Leinwand, L. A. (2015) miR-30 family microRNAs regulate myogenic differentiation and provide negative feedback on the microRNA pathway. *PloS one* **10**, e0118229

8. Eisenberg, I., Eran, A., Nishino, I., Moggio, M., Lamperti, C., Amato, A. A., Lidov, H. G., Kang, P. B., North, K. N., Mitrani-Rosenbaum, S., Flanigan, K. M., Neely, L. A., Whitney, D., Beggs, A. H., Kohane, I. S., and Kunkel, L. M. (2007) Distinctive patterns of microRNA expression in primary muscular disorders. *Proceedings of the National Academy of Sciences of the United States of America* **104**, 17016-17021

9. Zhang, J., Ying, Z. Z., Tang, Z. L., Long, L. Q., and Li, K. (2012) MicroRNA-148a promotes myogenic differentiation by targeting the ROCK1 gene. *The Journal of biological chemistry* **287**, 21093-21101

10. Kim, H. K., Lee, Y. S., Sivaprasad, U., Malhotra, A., and Dutta, A. (2006) Muscle-specific microRNA miR-206 promotes muscle differentiation. *The Journal of cell biology* **174**, 677-687

11. Rosenberg, M. I., Georges, S. A., Asawachaicharn, A., Analau, E., and Tapscott, S. J. (2006) MyoD inhibits Fstl1 and Utrn expression by inducing transcription of miR-206. *The Journal of cell biology* **175**, 77-85

12. Pardo, P. S., Hajira, A., Boriek, A. M., and Mohamed, J. S. (2017) MicroRNA-434-3p regulates age-related apoptosis through eIF5A1 in the skeletal muscle. *Aging* **9**, 1012-1029
